# Profiling of Lymphovascular Space Invasion in Cervical Cancer Revealed PI3K/Akt Signaling Pathway Overactivation and Heterogenic Tumor-Immune Microenvironments

**DOI:** 10.3390/life13122342

**Published:** 2023-12-14

**Authors:** Yeseul Choi, Yu Ando, Donghyeon Lee, Na Young Kim, Olive E. M. Lee, Junghwan Cho, Incheol Seo, Gun Oh Chong, Nora Jee-Young Park

**Affiliations:** 1 Graduate Program, Department of Biomedical Science, School of Medicine, Kyungpook National University, Daegu 41944, Republic of Korea; yeseul.choi830@knu.ac.kr (Y.C.); yuando@knu.ac.kr (Y.A.); lowellkids24@knu.ac.kr (D.L.); skduddl98@knu.ac.kr (N.Y.K.); olive@knu.ac.kr (O.E.M.L.); 2 Clinical Omics Institute, Kyungpook National University, Daegu 41405, Republic of Korea; joshua@knu.ac.kr (J.C.); iseo@knu.ac.kr (I.S.); 3 Department of Immunology, School of Medicine, Kyungpook National University, Daegu 41944, Republic of Korea; 4 Department of Obstetrics and Gynecology, Kyungpook National University Chilgok Hospital, Daegu 41404, Republic of Korea; 5 Department of Pathology, Kyungpook National University Chilgok Hospital, Daegu 41404, Republic of Korea

**Keywords:** cervical cancer, lymphovascular space invasion, RNA sequencing, PI3K/Akt signaling pathway, sustained angiogenesis, immune deconvolution, tumor immune microenvironment, TIME, regulatory T cells

## Abstract

Lymphovascular space invasion (LVSI) is the presence of tumor emboli in the endothelial-lined space at the tumor body’s invasive edge. LVSI is one of three Sedlis criteria components—a prognostic tool for early cervical cancer (CC)—essential for indicating poor prognosis, such as lymph node metastasis, distant metastasis, or shorter survival rate. Despite its clinical significance, an in-depth comprehension of the molecular mechanisms or immune dynamics underlying LVSI in CC remains elusive. Therefore, this study investigated tumor-immune microenvironment (TIME) dynamics of the LVSI-positive group in CC. RNA sequencing included formalin-fixed paraffin-embedded (FFPE) slides from 21 CC patients, and differentially expressed genes (DEGs) were analyzed. Functional analysis and immune deconvolution revealed aberrantly enriched PI3K/Akt pathway activation and a heterogenic immune composition with a low abundance of regulatory T cells (Treg) between LVSI-positive and LVSI-absent groups. These findings improve the comprehension of LSVI TIME and immune mechanisms, benefiting targeted LVSI therapy for CC.

## 1. Introduction

Lymphovascular space invasion (LVSI) is the presence of tumor emboli within dilated endothelium-lined spaces, such as lymphatic vessels or small capillaries at the invasive front of a tumor [1,2,3,4]. As a crucial initial step of the invasion–metastasis cascade, LSVI is an early indication of lymph node and other distant metastasis and poor prognosis, such as shorter recurrence-free survival (RFS) [4,5,6,7]. With such clinical significance, LVSI is a prominent prognostic factor of cancer in women, including endometrial, breast, and cervical cancers (CC) [5,8,9,10,11,12].

In particular, LVSI in CC is one of three Sedlis criteria—intermediate risk factors concerning early CC prognosis—along with a tumor size larger than 4 cm and deep stromal invasion [13,14,15,16,17]. As LVSI is an integral route that tumor cells disseminate, it guides physicians to inspect the possibility of local invasion and distant metastasis as well [18]. Furthermore, LVSI assists clinicians in surgical methods or patient management decisions, such as paraaortic lymphadenectomy, adjuvant pelvic radiation, or aggressive chemotherapy [16,19,20,21].

Despite its clinical significance, the specific molecular mechanisms or immune microenvironment dynamics underlying LVSI in CC remain poorly understood. While some studies have identified genomic alterations associated with LVSI, such as cancer cell angiogenesis or the enrichment of cell proliferation genes, these findings are predominantly observed in LVSI-positive cases of other cancer types, such as breast or colorectal cancers [22,23,24]. Limited research has explored the gene signatures or immune mechanisms of LVSI specifically in CC, such as studies conducted by Huang et al. [25] and He et al. [18]. Nonetheless, a comprehensive exploration of LVSI in CC is notably scarce within the existent literature, particularly concerning tumor-immune microenvironments (TIME). Therefore, the present study investigated TIME’s immune composition and dynamics within the LVSI-positive group in CC. Moreover, we sought a prominent immune marker that highly correlates with LVSI presence in CC to facilitate the detection of potential LVSI biomarkers and improve LVSI treatment.

## 2. Materials and Methods

### 2.1. Patient Selection

This study selected 21 patients who previously received radical hysterectomy for CC. Conventional pelvic lymphadenectomy was performed in a standard fashion, involving the removal of lymph nodes from external and internal iliac as well as obturator nodal stations. Low paraaortic lymphadenectomy added presacral, low paraaortic, and common iliac lymph nodes to conventional pelvic lymphadenectomy. Extended lymphadenectomy was defined as incorporation of superior and inferior gluteal lymph nodes with low paraaortic lymphadenectomy. Adjuvant concurrent chemoradiotherapy was administered for positive lymph nodes, parametrial invasion, or positive surgical margin, while adjuvant radiotherapy and chemotherapy were employed for intermediate risk factors.

Among the 21 patients, 15 had LVSI and were labeled as the LVSI-positive group (LVSI+), and the remaining 6 were considered the LVSI-absent group (LVSI−). All patients were staged per the revised 2009 International Federation of Gynecology and Obstetrics (FIGO) staging system. The Kyungpook National University Chilgok Hospital’s (KNUCH) ethics committee thoroughly reviewed the patient selection process following IRB protocol (IRB number approval number: 2020-10-003). T-tests were conducted for the variables “Age” and “Primary tumor size”, and Fisher’s exact tests were applied to the rest of the variables.

### 2.2. Pathological Processing and LVSI Detection

From surgically resected tissue of 21 CC patients, samples were stained with H&E on formalin-fixed paraffin-embedded (FFPE) slides. The available H&E slides, ranging from five to ten for each case, were then carefully reviewed by a gynecologic pathology specialist from KNUCH. LVSI was defined by the presence of tumor cells within endothelial-lined vascular/lymphatic spaces at the invasive front. Intratumoral LVSI was not considered. The slides were further immunostained with vascular endothelial markers CD31 and CD34 and lymphatic endothelial marker D2-40 for LVSI detection for cases requiring additional confirmation. Due to the spatial heterogeneity on the FFPE slides and the controversiality of quantification LVSI in CC, any identified focus of LVSI was considered as LVSI positive.

### 2.3. RNA Sequencing and LVSI DEGs

RNA was extracted from FFPE slides with the ReliaPrep™ FFPE Total RNA Miniprep System (Promega, Madison, WI, USA). Extracted RNA was generated into the library using the TruSeq RNA Exome Kit (Illumina, San Diego, CA, USA) and further sequenced with the Nova6000 platform (Illumina). Raw fastq data were quantified using the STAR [26] and RSEM [27], and differentially expressed gene (DEG) analysis was accomplished with DESeq2 [28]. DEGs with a threshold of an absolute log2 fold change ≥1 with adjusted *p*-value < 0.05 were considered significant. A volcano plot of the DEGs was plotted using the “ggplot” in R studio version 4.3.1 (https://posit.co/download/rstudio-desktop/) accessed on 10 May 2023.

### 2.4. Immune Pathways Enrichment Analysis with IMPAGT

The Immune Pathway and Gene Database (IMPAGT) [29] is an in-house immune database referenced from LM22 of CIBERSORT [30]. Unlike other immune databases that do not furnish comprehensive lists of genes associated with specific immune mechanisms, such as CIBERSORT, IMPAGT simultaneously provides immune pathways and their corresponding gene sets. Furthermore, IMPAGT offers insights into the involvement of individual genes in multiple immune pathways, enabling the interpretation of “one gene–many immune pathways” interconnections. Contrasting conventional “one gene–one pathway” analyses from other immune databases, this unique attribute allows researchers to view and interpret TIME from more holistic and integrative perspectives.

Intersection of LVSI DEGs with a 2534 IMPAGT gene set was performed through R studio. Overlapped LVSI DEGs were traced back on IMPAGT, and DEGs participating in each pathway were counted. Pathways with more than five involved DEGs were considered as significantly enriched TIME pathways by IMPAGT. After selecting enriched TIME pathways, the pathways were visualized with overlapping LVSI DEGs via KEGG Mapper [31]. The overlapped up-regulated DEGs were colored red.

### 2.5. Functional Analysis of Intersected LVSI DEGs with IMPAGT

For the functional analysis of LVSI DEGs that intersected with IMPAGT, the protein−protein interaction (PPI) network was reviewed via the STRING database [32], and gene ontology (GO) terms were analyzed on the DAVID database [33]. Among three GO terms (biological process [BP], cellular component, and molecular functions), BP terms were primarily investigated. Significant BP terms (*p*-value < 0.05) were further investigated through the STRING database.

### 2.6. Immune Microenvironment Estimation with Immune Deconvolution

The overall estimation of the 21 CC samples’ immune microenvironment was conducted using the R package “*Immunedeconv*”, which allows seven immune deconvolution methods: EPIC, xCell, TIMER, CIBERSORT, MCP Counter, and quantiseq [34]. The TPM values of the 21 CC samples were utilized as the input. Among the seven deconvolution tools, EPIC [35] and xCELL [36] results were further analyzed as they provide an “absolute score” of each sample, enabling inter-comparisons between the samples. EPIC provided seven immune cell types: B cells, cancer-associated fibroblasts, endothelial cells, macrophages, NK cells, T cell CD4+, and T cell CD8+. xCell provided 36 immune cell types, such as B cells, cancer-associated fibroblasts, endothelial cells, eosinophils, macrophages, macrophage M1, mast cells, and regulatory T cells (Tregs). K-means clustering was applied to the immune deconvolution result matrix of EPIC and xCell. The initial k was set as two to investigate whether immune composition grouping corresponded to the absence or presence of LVSI (LVSI+/−).

In order to investigate immune markers highly correlated with LVSI, point–biserial correlation—which measures the association between continuous numeric and binary categorical variables—was applied to xCell immune markers and LVSI. As xCell provided more immune cell types than EPIC, xCell results were utilized. Furthermore, all 36 xCell immune cell types were evaluated for their association with LVSI+ based on a univariate logistic regression model. The *t*-test was also performed to identify differences between LVSI+/− groups. All statistical analyses were conducted with the base R function in R studio.

## 3. Results

### 3.1. Clinicopathological Characteristics of the 21 Samples

Several clinical parameters for the 21 samples were collected and presented relative to LVSI status (Table 1). Compared with the LVSI− group, the LVSI+ group was primarily staged in later FIGO stages, such as IIA2 or IIB. Most samples with deep stromal invasion, parametrial invasion, and lymph node metastasis were from the LVSI+ group. Also, samples from the LVSI+ group received a broader extent of lymphadenectomy as well as adjuvant therapy compared with the LVSI− group.

### 3.2. Representative LVSI Detection

**Figure 1:**
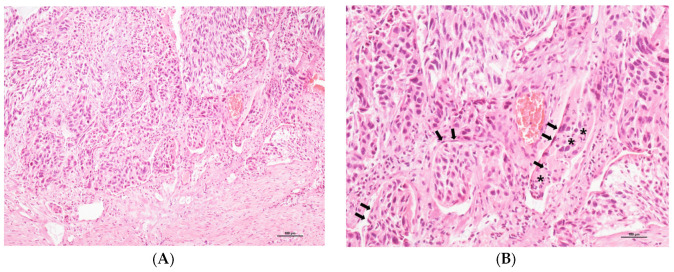
Representative microscopic features of LVSI (H&E stains; (**A**), ×100 magnification; (**B**), ×200). (**A**) Malignant cell clusters are identified within the lymphovascular spaces at the leading edge of invasive cervical cancer. (**B**) Tumor cell clusters are covered with endothelial cells (indicated by black arrows) and intermixed with inflammatory cells (indicated by black asterisks).

Representative microscopic features of LVSI are depicted in Figure 1 through H&E-stained slides.

### 3.3. Differentially Expressed Genes (DEGs)

**Figure 2:**
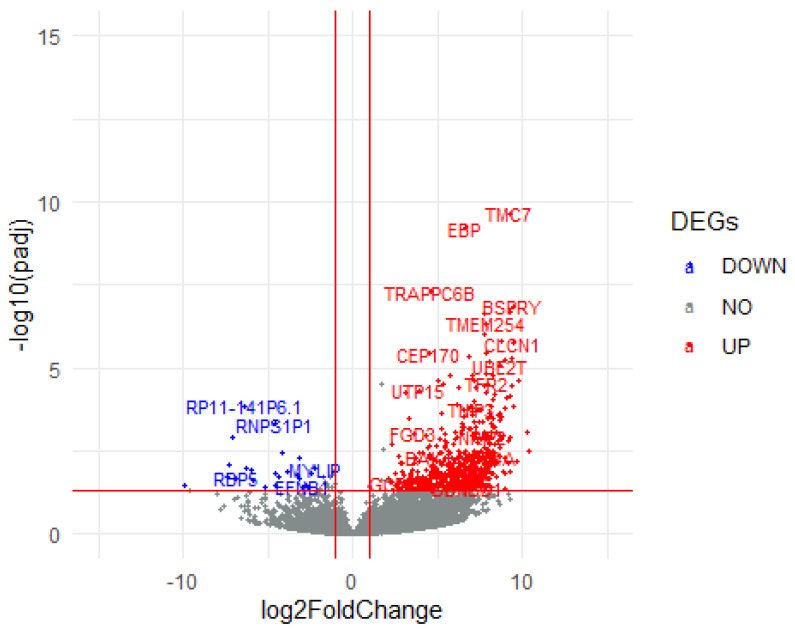
A volcano plot of LVSI+ DEGs. The right side of the volcano plot indicates up−regulated LVSI DEGs in red, and the left side depicts down-regulated LVSI DEGs in blue.

There were 734 DEGs between LVSI− and LVSI+ groups. Among the 734 DEGs, 708 were up-regulated, and the remaining 26 were down-regulated (Supplementary S1). A volcano plot of significant LVSI+ DEGs is presented in Figure 2.

### 3.4. Intersection of LVSI DEGs with IMPAGT Revealed PI3K/Akt Signaling Pathway Overactivation

In order to investigate the TIME dynamics of the LVSI+ group in CC, LVSI DEGs that overlapped with the IMPAGT gene set were further researched. Overall, 67 LVSI DEGs overlapped with IMPAGT; all overlapped with up-regulated LVSI DEGs, and none from down-regulated LVSI DEGs (Supplementary S2). Pathways with more than five DEGs from IMPAGT were considered significantly enriched and are presented in Table 2.

The most enriched pathway was “Pathway in Cancer” with 16 DEGs, followed by “PI3K/Akt Signaling Pathway” with 13 DEGs. The remaining enriched pathways were as follows: “Cytokine–Cytokine Receptor Interaction”, “Cell Adhesion Molecules”, “Hematopoietic Cell Lineage”, and “MAPK Signaling Pathway”.

**Figure 3:**
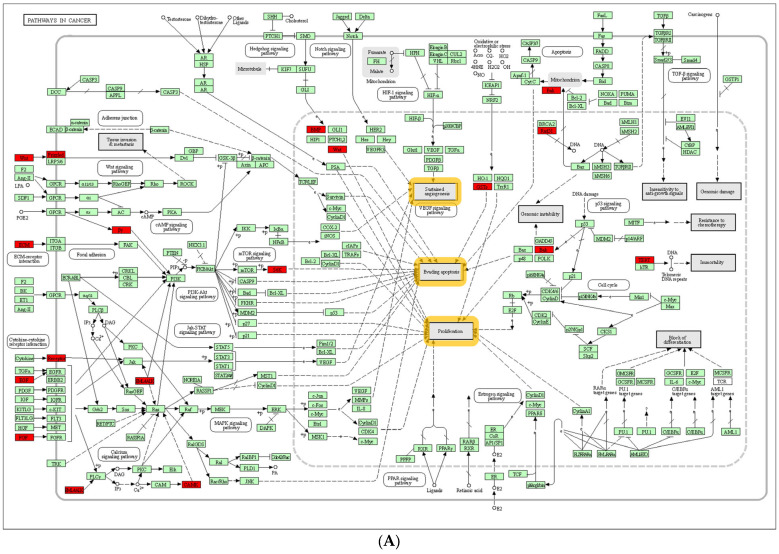

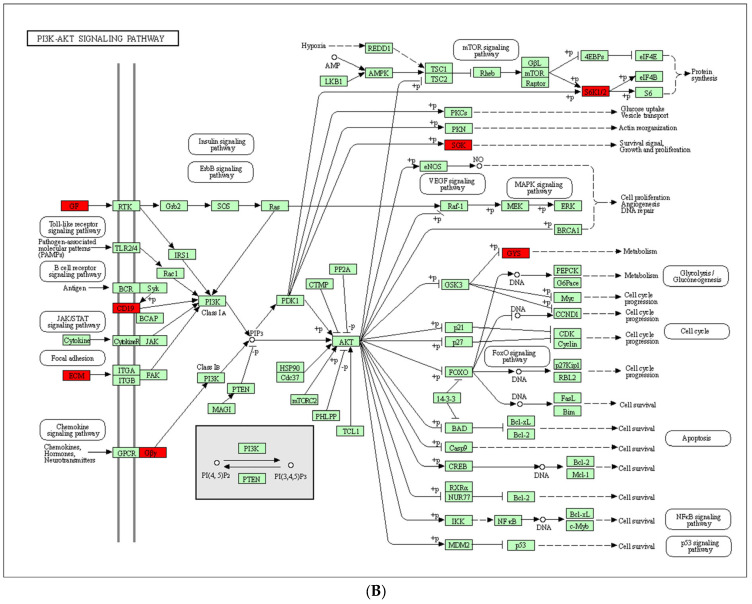
Enriched pathway visualization with KEGG Mapper. Up−regulated LVSI DEGs are highlighted in red. (**A**) “Pathway in Cancer”; all signals lead toward ‘Sustained Angiogenesis’, ‘Evading Apoptosis’, and ‘Proliferation’, which are highlighted in orange. (**B**) “PI3K−Akt Signaling Pathway”.

Pathway enrichment visualization via KEGG Mapper highlighted where overlapped DEGs participated and were activated in each pathway. DEGs in “Pathway in Cancer” were primarily related to receptor interactions on the cell membrane. Additionally, they were involved in signaling pathways, such as phosphoinositide 3-kinase (PI3K)/Akt, mitogen-activated protein kinase (MAPK), or vascular endothelial growth factor (VEGF) signaling pathways, which eventually directed toward “Proliferation”, “Evading Apoptosis”, and “Sustained Angiogenesis” (Figure 3A).

As the “PI3K/Akt Signaling Pathway” was the second most enriched pathway and part of the “Pathway in Cancer”, it was also visualized with KEGG Mapper (Figure 3B). Highlighted DEGs were mainly involved in pathways as growth factors or ECM receptors that activated PI3K/Akt signaling via Class 1A PI3K isoforms.

#### 3.4.1. Functional Analysis Revealed 67 LVSI-IMPAGT DEGs Are Related to Tumoral Angiogenesis in CC

The 67 LVSI DEGs overlapped with IMPAGT were further investigated on the DAVID database for BP terms; significantly identified BP terms are presented in Table 3. To examine gene interactions relative to BP functions, 67 DEGs were also examined on the STRING database. The functional analysis revealed that BP terms were predominantly related to tumoral angiogenesis in CC.

**Figure 4:**
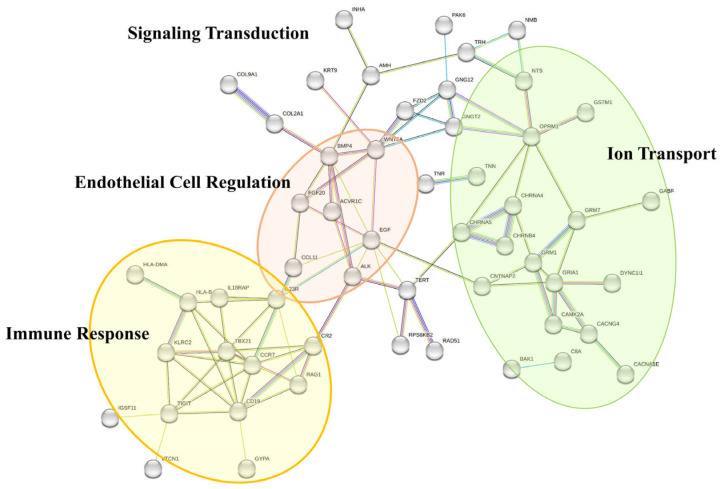
PPI network of the 67 LVSI−IMPAGT DEGs with their significant BP terms. While the overall PPI network was related to signal transduction, PPI subgroups tended to cluster in three representative BP traits: “Immune Response”, “Ion Transport”, and “Regulation of Endothelial Cells”.

The overall PPI network was linked to “Signaling Systems”, such as ‘Signal Transduction’ and ‘Cell–Cell Signaling’. Interestingly, the PPI network tended to cluster into three representative BP traits: “Immune Response”, “Ion Transport”, and “Regulation of Endothelial Cells” (Figure 4). First, genes positioned in the right half of the PPI network were primarily related to “Ion Transport” BP traits, such as “Ion Transmembrane Transport” or “Calcium Ion Transport”. In addition, the inner cluster was related to “Regulation of Endothelial Cell”, including “Positive Regulation of Endothelial Proliferation” and “Positive Regulation of Endothelial Cell Migration”. Lastly, genes located in the bottom left of the PPI network were largely related to “Immune Response”, as follows: “Positive Regulation of T cell-Mediated Cytotoxicity”, “Positive Regulation of Immune Response”, “Antigen Processing and Presentation”, “Adaptive immune response”, and “B cell differentiation”.

#### 3.4.2. Immune Deconvolution Exposed Heterogeneity in the Immune Composition of the LVSI+ Group with CC

**Figure 5:**
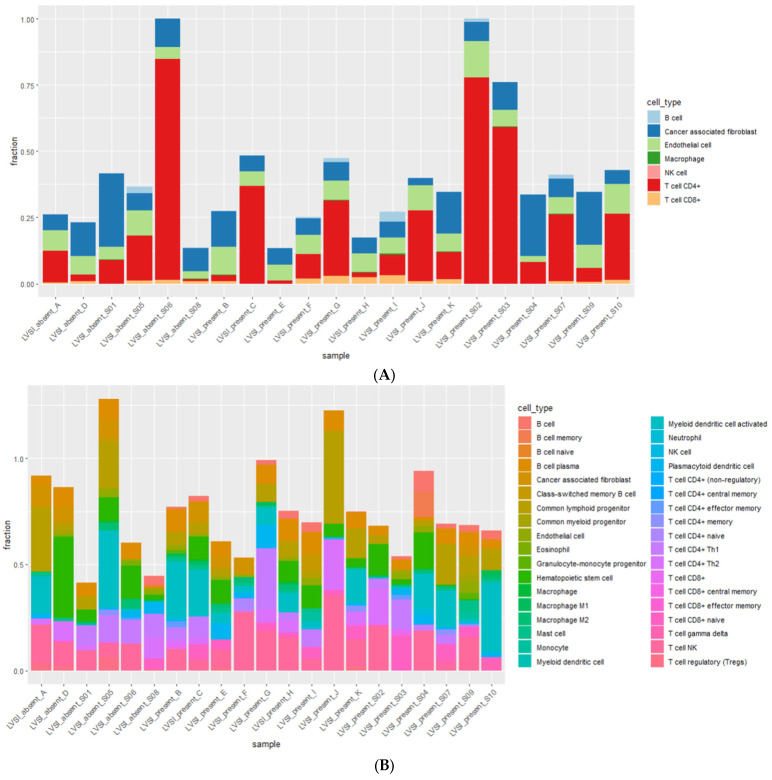
Immune deconvolution results revealed the overall immune composition of 21 CC patients. The *x*-axis indicates the overall 21 CC samples of the study, and the *y*-axis delineates each tool’s absolute immune deconvolution scores. (**A**) EPIC results with seven immune cell types, and (**B**) xCell results with thirty-six immune cell types.

First, immune deconvolution tools were employed to comprehend the overall immune composition of the 21 CC samples. EPIC results presented seven major immune cell types, including B, CD4+ T, and CD8+ T cell types (Figure 5A). xCell indicated 36 immune cell types with specific subsets, such as naïve B, memory B, Th 1 CD4+ T, and regulatory T cell types (Figure 5B). The overall immune composition results from EPIC and xCELL were clustered to investigate whether immune composition differed relative to LVSI, setting the initial k as two. The two clusters from both deconvolution results did not separate according to the clinical parameter of interest, LVSI+/−.

**Figure 6:**
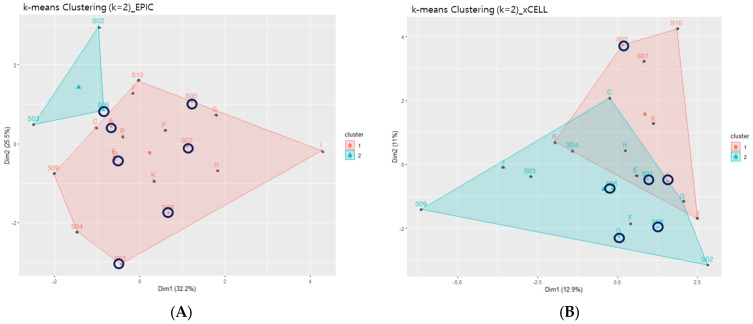
The k−means clustering plots of (**A**) EPIC and (**B**) xCELL immune deconvolution results. Black circles indicate LVSI− samples. Although the plots did not cluster relative to LVSI, the LVSI− group tended to cluster as a sub-group, indicating a similarity in immune compositions. On the other hand, the LVSI+ group was dispersed throughout the plot, revealing a more “heterogenic” immune composition of the LVSI+ group than the LVSI− group.

Although the EPIC results had a distinct separation of two clusters, it did not correlate with LVSI+/− (Figure 6A). However, when the location of samples from each LVSI+ and LVSI− group were examined, samples from the LVSI− group were primarily localized in the middle of cluster 2. Regarding xCell result clustering, two clusters overlapped (Figure 6B). However, these results revealed that most samples from the LVSI− group were a small sub-cluster within cluster 2. The adjacent locality of LVSI− samples indicates that the LVSI− group shares similar immune compositions.

On the other hand, samples from the LVSI+ group were dispersed throughout the plot, located in clusters 1 and 2 when compared with the LVSI− group. This plot dispersion delineates immune composition diversity in the LVSI+ group, contrasting the shared similarity of the LVSI− group. In short, the LVSI+ group in CC presented more “heterogeneity” of TIME composition than the LVSI− group.

#### 3.4.3. Regulatory T Cells as Primary Immune Markers Related to the LVSI+ Group within the Heterogenic Immune Composition of LVSI+ in CC

After examining the heterogenic immune composition of the LVSI+ group in CC, we investigated which immune markers highly correlated with LVSI+ parameters within such heterogeneity. As xCell provided more immune cell types compared with EPIC, xCell results were taken to examine the highly correlated immune markers with LVSI+. First, the point–biserial correlation was calculated between LVSI parameters and 36 immune cell types identified by xCell. A correlation greater than an absolute value of 0.35 was considered a significant immune cell marker related to the LVSI+ group.

**Figure 7:**
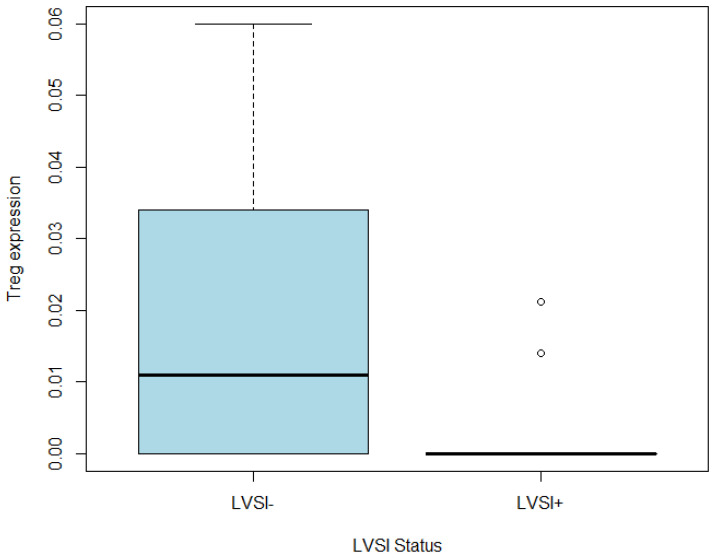
A boxplot of the low Treg abundance in the LVSI+ group. The *x*-axis represents LVSI+/− groups, and the *y*-axis depicts the abundance scores from xCell.

Three immune cell markers significantly correlated with LVSI+: regulatory T (Treg), eosinophil, and T cell CD8+ naïve cells (Supplementary S3). Among these immune markers, Tregs presented the strongest correlation with LVSI+ with a negative correlation of −0.50. The following *t*-test on Treg abundance score comparison between LVSI+/− groups, with a *p*-value of 0.07, indicated a proclivity of low Treg expression in the LVSI+ group (Figure 7). The *t*-test results on the rest of two immune markers were as follows: eosinophil (*p* = 0.33) and T cell CD8+ naïve cells (*p* = 0.03). Furthermore, in order to identify whether these immune markers can predict the presence of LVSI, the univariate regression analysis was performed on 36 xCell immune markers, and it revealed that Treg was the only significant immune marker related to LVSI+ (coefficient: −14.09, *p*-value < 0.02). These findings indicate that Treg is the predominant immune cell marker that presents less abundance in LVSI+ parameters among the heterogenic immune composition of LVSI+ in CC.

## 4. Discussion

This study investigated TIME and immune dynamics underlying LVSI in CC. TIME is a multifaceted architecture where the crosstalk and interplay of various cell types from tumors, stroma, ECM, and immune cells occur [37]. As dynamic reciprocal communication between cells in TIME switches and favors cancer progression, TIME interpretation and comprehension are critical. Therefore, this study elucidates the tumor-immune dynamics and mechanisms of LVSI in CC: the aberrantly enriched PI3K/Akt signaling pathway and heterogenic immune composition of LVSI in CC with low Treg abundance.

First, this study revealed a highly enriched activation of the PI3K/Akt signaling pathway in LVSI+ in CC. From the enriched immune pathway analysis, “Pathway in Cancer” was identified as the most enriched pathway. Several signaling pathways, such as the PI3K/Akt signaling pathway or MAPK signaling pathway, were involved in the mechanism responsible for the major cancer hallmarks proposed by Weinberg: “Proliferation”, “Avoiding Apoptosis,” and “Sustained Angiogenesis” [38]. As the PI3K/Akt signaling pathway was involved in “Pathway in Cancer” and was the second most enriched pathway from LVSI+ in CC, the PI3K/Akt signaling pathway was investigated further.

As a primary signaling pathway, the PI3K/Akt signaling pathway manifests various cellular processes, such as the cell cycle, proliferation, or apoptosis. With such engagement, the PI3K/Akt signaling pathway also participates in signaling cascades toward prominent cancer hallmarks. As previously mentioned, these markers include malignant cell proliferation or anti-apoptosis. Furthermore, the PI3K/Akt signaling pathway is also involved in sustained angiogenesis. In tumor cells, PI3K/Akt signaling pathway activation boosts VEGF secretion by hypoxia-inducible factor (HIF) dependent and independent mechanisms. Subsequently, this process increases the expression of angiogenic factors, such as VEGF, angiopoietins, or nitric oxide [39]. These angiogenic factors then augment vascular permeability that contributes to leaky vessels, sluggish blood flow, and interstitial pressure of the endothelial vessel, otherwise known as sustained angiogenesis [39,40]. Among three PI3K classes, the p110a isoform from class 1 PI3K is an essential stimulator of angiogenesis by regulating VEGF and dictating overall cancer progression [41,42,43,44,45].

The BP terms analyzed in this study, such as “Ion Transport” and “Regulation of Endothelial Cells”, further support and highlight the PI3K/Akt signaling pathway’s involvement in “Sustained Angiogenesis.” According to the literature, membrane potential (Vm) is depolarized in cancer cells compared with normal counterparts [46]. Moreover, dysregulated Vm is prevalent throughout all cancer progression stages and nearly all cancer hallmarks [47,48,49]. As Ca^2+^ transport was identified as a significant BP term, Ca^2+^ entry concerning angiogenesis was further investigated.

There are a few mechanisms that describe calcium entry with angiogenesis. Ca^2+^ enters the cytosol through Ca^2+^ permeable plasma membrane channels, such as transient receptor potential (TRP) or store-operated Ca^2+^ entry (SOCE) [50]. Under hypoxic tumor microenvironments (TME), tumor cells secrete growth factors or cytokines, including VEGF and epidermal growth factors (EGF). VEGF then combines with VEGF receptor 2, which allows Ca^2+^ entry via SOCE or TRP [51]. In particular, TRP is up-regulated in hypoxic cancer cell environments and is considered an “angiogenic switch” that facilitates angiogenic mechanisms.

For example, TRP subtypes—such as TRPC1 and TRPC4—encourage endothelial cell proliferation and migration. Similarly, TRPC3 and TRPC6 activation increases endothelial cell migration and permeability. Altogether, these subtypes enhance pro-angiogenic factor secretion, such as VEGF or EGF, that leads to blood capillary sprouts [50]. TRPC4 is related to increased intracellular Ca^2+^ influx and PI3K/Akt signaling pathway activation via VEGF [52,53]. As such, TRPC4 is an essential pro-angiogenic determinant that maintains the balance between normal and defective vasculatures [54]. Moreover, TRP also forms a complex with STIM1 and Orail of SOCE, allowing sustained Ca^2+^ influx that increases EMT, sustained angiogenesis, and poor prognosis [55].

This study also observed the “heterogeneity” of TIME of LVSI+ in CC. Although the clusters did not separate relative to LVSI status, k-means plots from the deconvolution results indicated a tendency that samples from the LVSI− group position in proximity. This proclivity implies shared immune composition characteristics among the LVSI− group. While LVSI− presented similar immune compositions, samples from the LVSI+ group were dispersed throughout the two clusters of the k-means plot. Such dispersion suggests a distinctive “heterogenic” TIME composition of the LVSI+ group in CC compared with the LVSI− group.

Regarding TIME heterogeneity, Fujikawa et al. proposed that TIME is more heterogeneous in aggressive tumors [8]. In addition, Karar et al. discovered that aberrant vasculature and active ECM remodeling corresponding to abnormal tumor blood vessels also influenced TIME heterogeneity [39]. Furthermore, Li et al. revealed extensive cellular heterogeneity in malignant CC epithelial cells via single-cell transcriptomics analysis [56]. Our findings corroborate such heterogeneity of LVSI+ in CC. Specifically, visualizing the PI3K/Akt signaling pathway with LVSI-IMPAGT DEGs highlighted that these DEGs primarily act as growth factors or ECM receptors that activate the PI3K/Akt signaling pathway, driving cell proliferation or angiogenesis. It elucidates atypical enrichment and alterations in PI3K/Akt signaling are associated with LVSI+ in CC. Such aberrant shifts within LVSI+ TIME may beget atypical immune cell expression, reshaping the TIME architecture of LVSI+ into a more heterogeneous landscape than the LVSI− group.

After acknowledging the heterogenic nature of the LVSI+ group’s immune composition, we aimed to identify immune markers that were highly correlated to the LVSI+ group within heterogeneous TIME. Point–biserial correlation and univariate regression revealed that low Treg abundance was the strongest correlated immune marker with LVSI+ among its heterogenic TIME architecture. A recent study by He et al. also supports this observation; they found a suppression of adaptive immunity in LVSI+ patients. Their findings presented lower abundance of active CD4 T cells, natural killer T cells, effector memory CD8 T cell, and Treg in the LVSI+ group in comparison with the LVSI− group [18].

Treg is widely known for its immune suppression in TIME [57,58,59]; however, this aspect is controversial [60,61,62]. For example, Hori et al. reviewed Foxp3+Tregs and debated Treg’s plasticity and heterogenetic role [63]. Considering this controversy and outcomes of Treg within TIME from various studies, Barua et al. stated the necessity for comprehending Treg spatially [64]. Specifically, Luznik et al. asserted Treg’s pro- and anti-angiogenic effects [65]. They further reported that Treg exerts opposing effects on angiogenic function relative to tissue or TIME characteristics in which Treg resides, stressing the importance of investigating Treg in a spatial- and context-specific manner [65].

The limitation of this study lies in its relatively small size and retrospective nature. The relatively smaller sample size of the LVSI− group compared with the LVSI+ group may hold the possibility of selection bias. However, it is crucial to highlight that the samples of the current study represent invaluable “real-world clinical samples”, which are relatively scarce in sample retrieval. Recognizing these concerns, ongoing efforts are focused on designing future studies with larger samples sizes and a more balanced composition. Integration of publicly available datasets related to LVSI+ in CC are actively explored to augment the sample numbers. Additionally, public datasets of LVSI+ in other cancer types are also being considered to validate the specificity of the finding in context of CC. This comprehensive approach would bolster robustness of the study while compensating current limitations. Furthermore, multi-omics approaches are being considered for upcoming studies, such as proteomics, metabolomics, spatial transcriptomics, and single-cell transcriptomics. These methods would further enable precise immune profiling and assist in the investigation and interpretation of specific TIME immune marker roles with localization information in LVSI+ in CC.

Recently, the FIGO made a revision and now requires precise documentation on the extent of LVSI when staging II endometrial cancer by counting the number of LVSI [66,67]. As such, the 2023 FIGO revision acknowledges and emphasizes LVSI’s enhanced prognostic value, but this revision has yet to be applied to CC. However, considering LSVI’s increased significance regarding cancer in women, this revision is expected soon. Given the limited research on LVSI+ in CC despite its increasing importance, the current findings significantly contribute to the medical understanding of LVSI+ in CC and further establish a foundational groundwork for comprehending LVSI+ in CC. Moreover, the findings further contribute to potential LVSI targeting therapies. For instance, the identification of an over-activated PI3K/Akt signaling pathway suggests a potential avenue for therapeutic explorations, such as PI3K inhibitor as EMT modulators to mitigate pathway effects. In addition, the “immune-hot” status of LVSI+ in CC, characterized by up-regulated immune signals and low Treg expression, suggests the feasibility of immunotherapy implementation. Modulating the immune response of LVSI+ in CC and the TIME architecture could inhibit further LVSI progression, enabling earlier invention against lymph node metastasis or distant organ metastasis. Further studies building upon these current findings would assist identifying more refined and detailed LVSI-targeted therapies.

## 5. Conclusions

LVSI, defined as the presence of tumor emboli in the endothelial-lined space at the tumor body’s invasive edge, serves as crucial prognostic criteria for CC with implications for surgical selection or patient management due to its association with poor prognosis. Despite its clinical significance, an in-depth comprehension of the molecular mechanisms and immune dynamics underlying LVSI in CC still remains elusive. Transcriptomic analysis of DEGs in LVSI+/− groups from 21 CC samples in the current study revealed the aberrant enrichment PI3K/Akt signaling pathway, particularly in relation to tumoral angiogenesis in CC. Furthermore, this study discovered the heterogeneity of the LVSI+ group’s immune composition in CC compared with the LVSI− group. In addition, low Treg expression was identified as a major correlated immune marker within the LVSI+ group in CC. These findings improve comprehension and open avenues for targeted treatments toward LVSI in CC.

## Figures and Tables

**Table 1:** Clinicopathological characteristics of the 21 samples in this study.

Variables	All (*n* = 21)	LVSI− (*n* = 6)	LVSI+ (*n* = 15)	*p*-Value
Age (years ± SD)	47.4 ± 11.0	46.8 ± 9.2	47.6 ± 11.3	0.880
FIGO stage (*n*, %)				0.586
IB1	11 (52.4)	4 (66.7)	7 (46.7)	
IB2	2 (9.5)	1 (16.7)	1 (6.7)	
IIA2	4 (19.0)	1 (16.7)	3 (20.0)	
IIB	4 (19.0)	0	4 (26.7)	
Histology				0.862
Squamous cell carcinoma	15 (71.4)	5 (83.3)	10 (66.7)	
Adenocarcinoma	5 (23.8)	1 (16.7)	4 (26.7)	
Adenosquamous carcinoma	1 (4.8)	0	1 (6.7)	
Primary tumor size (cm ± SD)	3.3 ± 1.6	2.7 ± 1.0	3.5 ± 2.0	0.367
Deep stromal invasion (*n*, %)	20 (95.2)	5 (83.3)	15 (100.0)	0.286
Parametrial invasion (*n*, %)	9 (42.9)	1 (16.7)	8 (53.3)	0.178
Positive vaginal margin (*n*, %)	2 (9.5)	1 (16.7)	1 (6.7)	0.500
Lymph node metastasis (*n*, %)	11 (52.4)	3 (50.0)	8 (53.3)	1.000
Lymphadenectomy				0.084
Pelvic lymphadenectomy	4 (19.0)	3 (50.0)	1 (6.7)	
Pelvic and low paraaortic lymphadenectomy	7 (33.3)	1 (16.7)	6 (40.0)	
Pelvic and extended lymphadenectomy	10 (47.6)	2 (33.3)	8 (53.3)	
Adjuvant therapy				0.835
Chemotherapy	5 (23.8)	1 (16.7)	4 (26.7)	
Radiotherapy	2 (9.5)	0	2 (13.3)	
Concurrent chemotherapy	10 (47.6)	2 (33.3)	8 (53.3)	
Recurrence (*n*, %)	6 (28.6)	4 (66.7)	2 (13.3)	0.031

For *p*-value, *t*-tests were conducted for the variables “Age” and “Primary tumor size”, and Fisher’s exact tests were applied to the rest of the variables. FIGO, International Federation of Gynecology and Obstetrics; (*n*, %) indicates the number and percentage of samples in each parameter; SD, standard deviation; LVSI+/−; lymphovascular space invasion positive/absent.

**Table 2:** Significantly enriched pathways from IMPAGT. The listed significantly enriched pathways are presented with their corresponding KEGG ID and the number of DEGs involved in each.

KEGG ID	IMPAGT Pathway	Number of DEGs
Hsa 05200	Pathway in Cancer	16
Hsa 04121	PI3K/Akt Signaling Pathway	13
Hsa 04060	Cytokine-Cytokine Receptor Interaction	9
Hsa 04514	Cell Adhesion Molecules	7
Hsa 04640	Hematopoietic Cell Lineage	5
Hsa 04010	MAPK Signaling Pathway	5

**Table 3:** Significant BP terms of 67 up-regulated LVSI DEGs overlapped with IMPAGT.

Group	GO: BP ID	BP Terms	*p*-Value
*Tumor*	GO: 0007165	Signal Transduction	<0.0001
	GO: 0051092	Positive Regulation of NF-kappa B Transcription Factor Activity	<0.001
	GO: 0034220	Ion Transmembrane Transport	0.001
	GO: 0001938	Positive Regulation of Endothelial Cell Proliferation	0.002
	GO: 0030334	Regulation of Cell Migration	0.007
	GO: 0007267	Cell–Cell Signaling	0.008
	GO: 0006468	Protein Phosphorylation	0.001
	GO: 0043410	Positive Regulation of MAPK Cascade	0.018
	GO: 0010595	Positive Regulation of Endothelial Cell Migration	0.022
	GO: 0032092	Positive Regulation of Protein Binding	0.028
	GO: 0006816	Calcium Ion Transport	0.037
*Immune*	GO: 0006955	Immune Response	<0.0001
	GO: 0001916	Positive Regulation of T Cell-Mediated Cytotoxicity	0.007
	GO: 0050778	Positive Regulation of Immune Response	0.007
	GO: 0030333	Antigen Processing and Presentation	0.012
	GO: 0002250	Adaptive Immune Response	0.028
	GO: 0030183	B Cell Differentiation	0.038

## Data Availability

Data are contained within the article and Supplementary Materials.

## Supplementary Materials

The following supporting information can be downloaded at: https://www.mdpi.com/article/10.3390/life13122342/s1, S1: LVSI DEGs; S2: 67 LVSI DEGs intersect with IMPAGT gene set; S3: Point-biserial correlation of three immune cell markers.

## References

[1] Sabel M.S. (2009). Reading the Pathology Report. Essentials of Breast Surgery.

[2] Tortorella L., Restaino S., Zannoni G.F., Vizzielli G., Chiantera V., Cappuccio S., Gioè A., La Fera E., Dinoi G., Angelico G. (2021). Substantial Lymph-Vascular Space Invasion (Lvsi) as Predictor of Distant Relapse and Poor Prognosis in Low-Risk Early-Stage Endometrial Cancer. J. Gynecol. Oncol..

[3] Schade G.R., Wright J.L., Lin D.W. (2016). Prognostic Significance of Positive Surgical Margins and Other Implications of Pathology Report. Prostate Cancer: Science and Clinical Practice.

[4] Oliver-Perez M.R., Padilla-Iserte P., Arencibia-Sanchez O., Martin-Arriscado C., Muruzabal J.C., Diaz-Feijóo B., Cabrera S., Coronado P., Martín-Salamanca M.B., Pantoja-Garrido M. (2023). Lymphovascular Space Invasion in Early-Stage Endometrial Cancer (LySEC): Patterns of Recurrence and Predictors. A Multicentre Retrospective Cohort Study of the Spain Gynecologic Oncology Group. Cancers.

[5] Aleskandarany M.A., Sonbul S.N., Mukherjee A., Rakha E.A. (2015). Molecular Mechanisms Underlying Lymphovascular Invasion in Invasive Breast Cancer. Pathobiology.

[6] Reig B., Moy L., Sigmund E.E., Heacock L. (2022). Biomarkers, Prognosis, and Prediction Factors.

[7] Alexander-Sefre F., Singh N., Ayhan A., Salveson H.B., Wilbanks G., Jacobs I.J. (2003). Detection of Tumour Lymphovascular Space Invasion Using Dual Cytokeratin and CD31 Immunohistochemistry. J. Clin. Pathol..

[8] Fujikawa H., Koumori K., Watanabe H., Kano K., Shimoda Y., Aoyama T., Yamada T., Hiroshi T., Yamamoto N., Cho H. (2020). The Clinical Significance of Lymphovascular Invasion in Gastric Cancer. In Vivo.

[9] Narayan K., Lin M.Y., Kondalsamy-Chennakesavan S., Mukhopadhyay A. (2021). Lymphovascular Space Invasion (LVSI)-Based Prognostic Clusters in Endometrial Cancer Patients Treated with Primary Surgery and Adjuvant Radiotherapy. Indian J. Gynecol. Oncol..

[10] Ørtoft G., Lausten-Thomsen L., Høgdall C., Hansen E.S., Dueholm M. (2019). Lymph-Vascular Space Invasion (LVSI) as a Strong and Independent Predictor for Non-Locoregional Recurrences in Endometrial Cancer: A Danish Gynecological Cancer Group Study. J. Gynecol. Oncol..

[11] Liu Y.L., Saraf A., Lee S.M., Zhong X., Hibshoosh H., Kalinsky K., Connolly E.P. (2016). Lymphovascular Invasion Is an Independent Predictor of Survival in Breast Cancer after Neoadjuvant Chemotherapy. Breast Cancer Res. Treat..

[12] Song Y.J., Shin S.H., Cho J.S., Park M.H., Yoon J.H., Jegal Y.J. (2011). The Role of Lymphovascular Invasion as a Prognostic Factor in Patients with Lymph Node-Positive Operable Invasive Breast Cancer. J. Breast Cancer.

[13] Creasman W.T., Kohler M.F. (2004). Is Lymph Vascular Space Involvement an Independent Prognostic Factor in Early Cervical Cancer?. Gynecol. Oncol..

[14] Morice P., Piovesan P., Rey A., Atallah D., Haie-Meder C., Pautier P., Sideris L., Pomel C., Duvillard P., Castaigne D. (2003). Prognostic Value of Lymphovascular Space Invasion Determined with Hematoxylin-Eosin Staining in Early Stage Cervical Carcinoma: Results of a Multivariate Analysis. Ann. Oncol..

[15] Li X., Xu C., Yu Y., Guo Y., Sun H. (2021). Prediction of Lymphovascular Space Invasion Using a Combination of Tenascin-C, Cox-2, and PET/CT Radiomics in Patients with Early-Stage Cervical Squamous Cell Carcinoma. BMC Cancer.

[16] Du W., Wang Y., Li D., Xia X., Tan Q., Xiong X., Li Z. (2021). Preoperative Prediction of Lymphovascular Space Invasion in Cervical Cancer With Radiomics –Based Nomogram. Front. Oncol..

[17] Park J.Y., Chong G.O., Park J.Y., Chung D., Lee Y.H., Lee H.J., Hong D.G., Han H.S., Lee Y.S. (2020). Tumor Budding in Cervical Cancer as a Prognostic Factor and Its Possible Role as an Additional Intermediate-Risk Factor. Gynecol. Oncol..

[18] He X. (2022). Bioinformatics Analysis of the Primary Molecular Mechanism in Lymphatic Vascular Space Invasion and Parametrial Invasion of Cervical Cancer. Res. Sq..

[19] Santoro A., Inzani F., Angelico G., Arciuolo D., Bragantini E., Travaglino A., Valente M., D’Alessandris N., Scaglione G., Sfregola S. (2023). Recent Advances in Cervical Cancer Management: A Review on Novel Prognostic Factors in Primary and Recurrent Tumors. Cancers.

[20] Zhang W., He W., Shi Y., Zhao J., Liu S., Zhang F., Yang J., Xie C., Zhang Y. (2017). Aberrant TIMELESS Expression Is Associated with Poor Clinical Survival and Lymph Node Metastasis in Early-Stage Cervical Carcinoma. Int. J. Oncol..

[21] Pol F.J.M., Zusterzeel P.L.M., Van Ham M.A.P.C., Kuijpers D.A.T., Bulten J., Massuger L.F.A.G. (2015). Satellite Lymphovascular Space Invasion: An Independent Risk Factor in Early Stage Cervical Cancer. Gynecol. Oncol..

[22] Kariri Y.A., Aleskandarany M.A., Joseph C., Kurozumi S., Mohammed O.J., Toss M.S., Green A.R., Rakha E.A. (2020). Molecular Complexity of Lymphovascular Invasion: The Role of Cell Migration in Breast Cancer as a Prototype. Pathobiology.

[23] Asaoka M., Patnaik S.K., Zhang F., Ishikawa T., Takabe K. (2020). Lymphovascular Invasion in Breast Cancer Is Associated with Gene Expression Signatures of Cell Proliferation but Not Lymphangiogenesis or Immune Response. Breast Cancer Res. Treat..

[24] Jiang H.H., Zhang Z.Y., Wang X.Y., Tang X., Liu H.L., Wang A.L., Li H.G., Tang E.J., Lin M. (2019). Bin Prognostic Significance of Lymphovascular Invasion in Colorectal Cancer and Its Association with Genomic Alterations. World J. Gastroenterol..

[25] Huang L., Zheng M., Zhou Q.M., Zhang M.Y., Jia W.H., Yun J.P., Wang H.Y. (2011). Identification of a Gene-Expression Signature for Predicting Lymph Node Metastasis in Patients with Early Stage Cervical Carcinoma. Cancer.

[26] Dobin A., Davis C.A., Schlesinger F., Drenkow J., Zaleski C., Jha S., Batut P., Chaisson M., Gingeras T.R. (2013). STAR: Ultrafast Universal RNA-Seq Aligner. Bioinformatics.

[27] Li B., Dewey C.N. (2014). RSEM: Accurate Transcript Quantification from RNA-Seq Data with or without a Reference Genome. Bioinformatics the Impact of Accurate Quantification on Proteomic and Genetic Analysis and Research.

[28] Love M.I., Huber W., Anders S. (2014). Moderated Estimation of Fold Change and Dispersion for RNA-Seq Data with DESeq2. Genome Biol..

[29] Choi Y., Park N.J.Y., Le T.M., Lee E., Lee D., Nguyen H.D.T., Cho J., Park J.Y., Han H.S., Chong G.O. (2022). Immune Pathway and Gene Database (IMPAGT) Revealed the Immune Dysregulation Dynamics and Overactivation of the PI3K/Akt Pathway in Tumor Buddings of Cervical Cancer. Curr. Issues Mol. Biol..

[30] Chen B., Khodadoust M.S., Liu C.L., Newman A.M., Alizadeh A.A. (2018). Profiling Tumor Infiltrating Immune Cells with CIBERSORT. Methods Mol. Biol..

[31] Kanehisa M., Sato Y. (2020). KEGG Mapper for Inferring Cellular Functions from Protein Sequences. Protein Sci..

[32] Szklarczyk D., Kirsch R., Koutrouli M., Nastou K., Mehryary F., Hachilif R., Gable A.L., Fang T., Doncheva N.T., Pyysalo S. (2023). The STRING Database in 2023: Protein-Protein Association Networks and Functional Enrichment Analyses for Any Sequenced Genome of Interest. Nucleic Acids Res..

[33] Huang D.W., Sherman B.T., Tan Q., Collins J.R., Alvord W.G., Roayaei J., Stephens R., Baseler M.W., Lane H.C., Lempicki R.A. (2007). The DAVID Gene Functional Classification Tool: A Novel Biological Module-Centric Algorithm to Functionally Analyze Large Gene Lists. Genome Biol..

[34] Sturm G., Finotello F., List M. (2020). Immunedeconv: An R Package for Unified Access to Computational Methods for Estimating Immune Cell Fractions from Bulk RNA-Sequencing Data. Methods Mol. Biol..

[35] Racle J., de Jonge K., Baumgaertner P., Speiser D.E., Gfeller D. (2017). Simultaneous Enumeration of Cancer and Immune Cell Types from Bulk Tumor Gene Expression Data. Elife.

[36] Aran D., Hu Z., Butte A.J. (2017). XCell: Digitally Portraying the Tissue Cellular Heterogeneity Landscape. Genome Biol..

[37] Fu T., Dai L.J., Wu S.Y., Xiao Y., Ma D., Jiang Y.Z., Shao Z.M. (2021). Spatial Architecture of the Immune Microenvironment Orchestrates Tumor Immunity and Therapeutic Response. J. Hematol. Oncol..

[38] Hanahan D., Weinberg R.A. (2011). Hallmarks of Cancer: The next Generation. Cell.

[39] Karar J., Maity A. (2011). PI3K/AKT/MTOR Pathway in Angiogenesis. Front. Mol. Neurosci..

[40] Garrido M.P., Torres I., Vega M., Romero C. (2019). Angiogenesis in Gynecological Cancers: Role of Neurotrophins. Front. Oncol..

[41] Jiang B.H., Liu L.Z. (2008). PI3K/PTEN Signaling in Tumorigenesis and Angiogenesis. Biochim. Biophys. Acta Proteins Proteom..

[42] Okkenhaug K., Graupera M., Vanhaesebroeck B. (2016). Targeting PI3K in Cancer: Impact on Tumor Cells, Their Protective Stroma, Angiogenesis, and Immunotherapy. Cancer Discov..

[43] Soler A., Serra H., Pearce W., Angulo A., Guillermet-Guibert J., Friedman L.S., Viñals F., Gerhardt H., Casanovas O., Graupera M. (2013). Inhibition of the P110α Isoform of PI 3-Kinase Stimulates Nonfunctional Tumor Angiogenesis. J. Exp. Med..

[44] Soler A., Angulo-Urarte A., Graupera M. (2015). PI3K at the Crossroads of Tumor Angiogenesis Signaling Pathways. Mol. Cell. Oncol..

[45] Graupera M., Guillermet-Guibert J., Foukas L.C., Phng L.K., Cain R.J., Salpekar A., Pearce W., Meek S., Millan J., Cutillas P.R. (2008). Angiogenesis Selectively Requires the P110α Isoform of PI3K to Control Endothelial Cell Migration. Nature.

[46] Yang M., Brackenbury W.J. (2013). Membrane Potential and Cancer Progression. Front. Physiol..

[47] Djamgoz M.B.A., Coombes R.C., Schwab A. (2014). Ion Transport and Cancer: From Initiation to Metastasis. Philos. Trans. R. Soc. B Biol. Sci..

[48] Fiorio Pla A., Munaron L. (2014). Functional Properties of Ion Channels and Transporters in Tumour Vascularization. Philos. Trans. R. Soc. B Biol. Sci..

[49] Andersen A.P., Moreira J.M.A., Pedersen S.F. (2014). Interactions of Ion Transporters and Channels with Cancer Cell Metabolism and the Tumour Microenvironment. Philos. Trans. R. Soc. B Biol. Sci..

[50] Arcangeli A., Crociani O., Bencini L. (2014). Interaction of Tumour Cells with Their Microenvironment: Ion Channels and Cell Adhesion Molecules. A Focus on Pancreatic Cancer. Philos. Trans. R. Soc. B Biol. Sci..

[51] Prevarskaya N., Ouadid-Ahidouch H., Skryma R., Shuba Y. (2014). Remodelling of Ca^2+^ Transport in Cancer: How It Contributes to Cancer Hallmarks?. Philos. Trans. R. Soc. B Biol. Sci..

[52] Moccia F., Negri S., Shekha M., Faris P., Guerra G. (2019). Endothelial Ca^2+^ Signaling, Angiogenesis and Vasculogenesis: Just What It Takes to Make a Blood Vessel. Int. J. Mol. Sci..

[53] Bong A.H.L., Monteith G.R. (2018). Calcium Signaling and the Therapeutic Targeting of Cancer Cells. Biochim. Biophys. Acta Mol. Cell Res..

[54] Martial S. (2016). Involvement of Ion Channels and Transporters in Carcinoma Angiogenesis and Metastasis. Am. J. Physiol. Cell Physiol..

[55] Jones C.A., Hazlehurst L.A. (2021). Role of Calcium Homeostasis in Modulating Emt in Cancer. Biomedicines.

[56] Li C., Wu H., Guo L., Liu D., Yang S., Li S., Hua K. (2022). Single-Cell Transcriptomics Reveals Cellular Heterogeneity and Molecular Stratification of Cervical Cancer. Commun. Biol..

[57] Litwin T.R., Irvin S.R., Chornock R.L., Sahasrabuddhe V.V., Stanley M., Wentzensen N. (2021). Infiltrating T-Cell Markers in Cervical Carcinogenesis: A Systematic Review and Meta-Analysis. Br. J. Cancer.

[58] Facciabene A., Motz G.T., Coukos G. (2012). T-Regulatory Cells: Key Players in Tumor Immune Escape and Angiogenesis. Cancer Res..

[59] Visser J., Nijman H.W., Hoogenboom B.N., Jager P., Van Baarle D., Schuuring E., Abdulahad W., Miedema F., Van Der Zee A.G., Daemen T. (2007). Frequencies and Role of Regulatory T Cells in Patients with (Pre)Malignant Cervical Neoplasia. Clin. Exp. Immunol..

[60] Galli F., Aguilera J.V., Palermo B., Markovic S.N., Nisticò P., Signore A. (2020). Relevance of Immune Cell and Tumor Microenvironment Imaging in the New Era of Immunotherapy. J. Exp. Clin. Cancer Res..

[61] Zhang Y., Lazarus J., Steele N.G., Yan W., Lee H.J., Nwosu Z.C., Halbrook C.J., Menjivar R.E., Kemp S.B., Sirihorachai V.R. (2020). Regulatory T-Cell Depletion Alters the Tumor Microenvironment and Accelerates Pancreatic Carcinogenesis. Cancer Discov..

[62] Pacella I., Piconese S. (2019). Immunometabolic Checkpoints of Treg Dynamics: Adaptation to Microenvironmental Opportunities and Challenges. Front. Immunol..

[63] Hori S. (2011). Regulatory T Cell Plasticity: Beyond the Controversies. Trends Immunol..

[64] Barua S., Fang P., Sharma A., Fujimoto J., Wistuba I., Rao A.U.K., Lin S.H. (2018). Spatial Interaction of Tumor Cells and Regulatory T Cells Correlates with Survival in Non-Small Cell Lung Cancer. Lung Cancer.

[65] Lužnik Z., Anchouche S., Dana R., Yin J. (2020). Regulatory T Cells in Angiogenesis. J. Immunol..

[66] Zheng W. (2023). Molecular Classification of Endometrial Cancer and the 2023 FIGO Staging: Exploring the Challenges and Opportunities for Pathologists. Cancers.

[67] Berek J.S., Matias-Guiu X., Creutzberg C., Fotopoulou C., Gaffney D., Kehoe S., Lindemann K., Mutch D., Concin N., Berek J.S. (2023). FIGO Staging of Endometrial Cancer: 2023. Int. J. Gynecol. Obstet..

