# Supplementary material for: Profiling of Lymphovascular Space Invasion in Cervical Cancer Revealed PI3K/Akt Signaling Pathway Overactivation and Heterogenic Tumor-Immune Microenvironments
Source: Life (Basel). 2023 Dec 14;13(12):2342. doi: 10.3390/life13122342 (PMC10744523; doi:10.3390/life13122342)
Supplement: Supplementary file 1 [file life-13-02342-s001.zip › Supplement S3. Point-biserial correlation of three immune cell markers.pdf]

Immune Marker	Point-biserial Correlation
Regulatory Tcell (Treg	-0.5025989
Eosinophil	-0.3515399
T cell CD8+ Naïve cell	0.3615544

Supplement S3. Point-biserial correlation which calculates correlation between numeric variables (expression of Immune cell marker of xCELL) and categorical variable (LVSI) was calculated.
